# Associations Between Individual Differences in Mathematical Competencies and Surface Anatomy of the Adult Brain

**DOI:** 10.3389/fnhum.2020.00116

**Published:** 2020-03-27

**Authors:** Alexander E. Heidekum, Stephan E. Vogel, Roland H. Grabner

**Affiliations:** Educational Neuroscience, Institute of Psychology, University of Graz, Graz, Austria

**Keywords:** right superior temporal gyrus, right central sulcus, left parieto-occipital sulcus, surface-based morphometry, mathematics

## Abstract

Previously conducted structural magnetic resonance imaging (MRI) studies on the neuroanatomical correlates of mathematical abilities and competencies have several methodological limitations. Besides small sample sizes, the majority of these studies have employed voxel-based morphometry (VBM)—a method that, although it is easy to implement, has some major drawbacks. Taking this into account, the current study is the first to investigate in a large sample of typically developed adults the associations between mathematical abilities and variations in brain surface structure by using surface-based morphometry (SBM). SBM is a method that also allows the investigation of brain morphometry by avoiding the pitfalls of VBM. Eighty-nine young adults were tested with a large battery of psychometric tests to measure mathematical competencies in four different areas: (1) simple arithmetic; (2) complex arithmetic; (3) higher-order mathematics; and (4) numerical intelligence. Also, we asked participants for their mathematics grades for their final school exams. Inside the MRI scanner, we collected high-resolution T1-weighted anatomical images from each subject. SBM analyses were performed with the computational anatomy toolbox (CAT12) and indices for cortical thickness, for cortical surface complexity, for gyrification, and sulcal depth were calculated. Further analyses revealed associations between: (1) the cortical surface complexity of the right superior temporal gyrus and numerical intelligence; (2) the depth of the right central sulcus and adults’ ability to solve complex arithmetic problems; and (3) the depth of the left parieto-occipital sulcus and adults’ higher-order mathematics competence. Interestingly, no relationships with previously reported brain regions were observed, thus, suggesting the importance of similar research to confirm the role of the brain regions found in this study.

## Introduction

Mathematical competencies are one of the key cognitive abilities in our modern societies, and they are crucial for our profession as well as social development (Parsons and Bynner, 2005). To date, an extensive number of neuroimaging studies have investigated the cognitive architecture of mathematical cognition. However, these studies have mainly focused on neurofunctional aspects of mathematical competencies, whereas literature on their neuroanatomical correlates is scarce. Especially, the surface characteristics of the gray matter (GM; i.e., cortical surface complexity, cortical thickness, gyrification, and sulcal depth) and their relationships with mathematical competencies have been widely ignored. The current study aimed to shed light on this aspect.

Numerous functional magnetic resonance imaging (fMRI) studies have demonstrated that numerical and mathematical cognition is associated with the activation of various frontal (e.g., ventral- and dorsolateral prefrontal cortex) and parietal brain regions (e.g., inferior and superior parietal cortex; for a review see Menon, 2014; for a meta-analysis see Arsalidou and Taylor, 2011). Also, it has been shown that the recruitment of these brain regions depends on the involved cognitive processes (e.g., quantity processing, arithmetic problem solving, etc.; Dehaene et al., 2003; Arsalidou and Taylor, 2011). For instance, while activation within the intraparietal sulcal (IPS) is thought to reflect quantity-based processes (Dehaene et al., 2003; Wilkey et al., 2017; Vogel et al., 2017a; or for a recent meta-analysis see Sokolowski et al., 2017), the controlled retrieval of a solution to a given arithmetic problem seems to rely on brain regions such as the inferior frontal gyrus (IFG), the supramarginal gyrus (SMG) and the angular gyrus (AG; Delazer et al., 2003, 2005; Ischebeck et al., 2006, 2009; Grabner et al., 2009; Klein et al., 2013, 2014; Menon, 2014; Peters and De Smedt, 2018; Heidekum et al., 2019). Furthermore, neuroimaging data indicate that activity in this network is also modulated by individual differences (e.g., Grabner et al., 2007; De Smedt and Gilmore, 2011; Berteletti et al., 2014). For instance, Grabner et al. (2007) compared two groups of young adults with different levels of mathematical competence who solved single-digit and multi-digit multiplication problems. The authors showed that individuals with higher mathematical competence more strongly activate the left AG while solving easy and more difficult multiplication problems. Grabner et al. (2007) concluded that mathematically more (compared to less) competent individuals rely more strongly on language-mediated processes (in particular fact retrieval) during arithmetic problem-solving.

In contrast to the large number of neurofunctional studies, only a few studies have used high-resolution structural MRI to examine how individual differences in brain morphology relate to variations in mathematical abilities and competencies (e.g., Isaacs et al., 2001; Aydin et al., 2007; Han et al., 2008, 2013; Rotzer et al., 2008; Rykhlevskaia et al., 2009; Lubin et al., 2013; Ranpura et al., 2013; Starke et al., 2013; Supekar et al., 2013; Cappelletti and Price, 2014; Evans et al., 2015; Price et al., 2016; Wilkey et al., 2018; Moreau et al., 2019). Many of those studies have focused on the comparison between dyscalculic individuals and controls (Han et al., 2008; Rotzer et al., 2008; Rykhlevskaia et al., 2009; Ranpura et al., 2013; Starke et al., 2013; Cappelletti and Price, 2014). For instance, it has been shown that dyscalculic adults have less GM volume in the right parietal cortex compared to controls (Cappelletti and Price, 2014) and that children with low mathematical skills relative to gender and age-matched controls show structural differences in the bilateral parietal lobes, right frontal lobe, and left occipital/parietal lobe (Han et al., 2008). These findings suggest an overlap of brain regions found in structural MRI studies and those reported in functional neuroimaging studies.

There are only a few studies (Supekar et al., 2013; Price et al., 2016; Wilkey et al., 2018) that investigated the neuroanatomical correlates of mathematics performance in typically developed individuals. However, most of these studies were conducted with children. For instance, Price et al. (2016) assessed the longitudinal and concurrent relations between GM volume and mathematical performance in a group of 50 children. GM volume maps derived from anatomical scans collected at the end of 1st grade and 2nd grade were related to performance on a standardized math test (i.e., Woodcock-Johnson III Tests of Achievement Calculation and Applied Problems). They found that left IPS GM volume at the end of 1st grade was positively related to math competence at the end of 2nd grade. Additionally, a positive association between GM volume in the same brain region at the end of 2nd grade and children’s concurrent math competence was observed. Wilkey et al. (2018) measured children’s performance in grade-level mathematics by a state-wide, school-based test of math achievement. They reported positive associations between differences in GM volume of the left and right hippocampal formations (including the hippocampus proper, entorhinal cortex, and subiculum) as well as the right IFG and children’s performance in grade-level mathematics.

Previous structural MRI studies on the neuroanatomical correlates of mathematical abilities and competencies have several methodological limitations. As highlighted above, the large majority of studies have involved children with mathematical learning disabilities (e.g., Han et al., 2013; Starke et al., 2013). Thus, they do not allow reliable inferences to typically developed populations because we cannot assume that neuroanatomical mechanisms that distinguish dyscalculic individuals from controls are the same that underlie individual differences in (typically developing) mathematical competence. Furthermore, we do not know whether the neuroanatomical mechanisms important during development are still playing a role in adults. Another methodological problem is the number of participants that were under investigation. Many findings are based on small sample sizes (Rotzer et al., 2008; Han et al., 2013), which is associated with various problems, such as low statistical power (Button et al., 2013) or an inflated false discovery rate (Colquhoun, 2014). Finally, most of these studies employed voxel-based morphometry (VBM) to investigate relationships between GM density and mathematical competencies. VBM is a technique that remains extremely popular because it is highly automated and therefore quick and easy to use. However, VBM also has been associated with important limitations in recent years. For example, recent studies have shown that differences in methodological choices like the registration algorithm (Peelle et al., 2012), or changes of user-specified parameters such as the smoothing kernel size (Henley et al., 2010) can lead to inconsistent VBM results. Furthermore, the interpretation of VBM findings is often difficult because the results may be driven by differences in cortical thickness, surface area (SA), cortical volume, and folding or any combination of these measures (Voets et al., 2008; Hutton et al., 2009).

A currently very promising alternative to VBM is surface-based morphometry (SBM), which also allows the investigation of brain morphometry. However, SBM uses brain surface meshes for spatial registration, which increases the accuracy of brain registration compared to mere volume-based registration (Desai et al., 2005). Further, it allows the calculation of additional measures of the neocortical surface structure, namely: cortical thickness (Dahnke et al., 2013), cortical surface complexity (quantification of the spatial frequency of gyrification and fissuration of the brain surface; Yotter et al., 2011), gyrification (Luders et al., 2006) and sulcal depth (Yotter et al., 2011). Due to these and further advantages, SBM has been frequently used in recent years, and a growing body of evidence suggests that cortical surface measures are particularly informative for individual differences in intellectual abilities (e.g., Geschwind and Rakic, 2013). For instance, longitudinal changes in cortical thickness have been related to variations in intelligence in children (Schnack et al., 2015) and a pronounced thickening of specific areas of the neocortex has been related to intellectual abilities in adulthood (Brans et al., 2010).

Only one study has used SBM in mathematical cognition so far. Moreau et al. (2019) investigated relationships between volumetric and surface characteristics of GM and dyslexia, dyscalculia and comorbid manifestation of both. They collected MRI data from four different groups of adults (i.e., dyslexic, dyscalculic, comorbid and control) and performed VBM as well as SBM analyses. By using Bayesian methods, Moreau et al. (2019) did not find any evidence for group differences in GM volume or any surface characteristics (i.e., cortical thickness, gyrification, sulcal depth, or cortical complexity). Therefore, the authors concluded that GM differences associated with these developmental disorders are not as reliable as previously suggested. However, these results were based on relatively small group samples (*N* = 12) and do not allow inferences to individual differences in typically developed populations.

Against this background, the current study aimed to investigate how structural variations in GM characteristics relate to individual differences in mathematical competencies in adults. Limitations of previous work were taken into account when designing the current study. First of all, to increase the power of the current study and the generalizability of its results, we collected data from a comparably large adult sample consisting of typically developed individuals. Thus, we attempted to identify neuroanatomical correlates of mathematical competencies in a population that has been underrepresented in previous studies. This will provide important insights into how the human brain mediates individual differences in mathematical cognition. Second, since previous studies showed that the neural correlates of mathematical competence depend on the mathematical demand (Price et al., 2016; Wilkey et al., 2018), we examined a broad range of mathematical competencies (e.g., arithmetic, higher-order mathematics) assessed using different instruments. Through the use of a large sample and different competence measures, we can investigate whether individual differences in mathematical competencies are related to a few brain regions of the classical model of mathematical cognition—as suggested by Dehaene et al. (2003)—or whether a more complex picture emerges. Finally, to avoid the above-described pitfalls of VBM we applied SBM to examine differences in brain surface structure (i.e., cortical thickness, cortical surface complexity, gyrification index, and sulcal depth).

## Materials and Methods

### Participants

Four-hundred and twenty-five German-speaking adults (female: *N* = 271; age: *M* = 23.13, *SD* = 5.58) participated in a larger behavioral test session, in which several psychometric measures were collected. Participants were recruited *via* social media, emails, and flyers. The majority of participants were enrolled as students at the University of Graz. All participants gave written informed consent before participation and received feedback regarding their intellectual abilities after testing as an incentive for taking part in the study.

From this pool, participants were recruited for two subsequent fMRI studies, in which, among other data, neurostructural images were collected. Both neuroimaging studies were carried out independently from each other. For the first neuroimaging study participants were randomly selected whereas in the second neuroimaging study participants were selected based on their arithmetic competencies (i.e., participants with lower and higher arithmetic competencies), which were measured in the behavioral test session. For both studies, all participants gave written informed consent before participation and were compensated with a minimum of € 20. The experimental procedure of both studies was approved by the ethics committee at the University of Graz, Austria.

For the current study, only participants from whom both data from the behavioral test session and neurostructural data were collected were included. In total 101 full data sets (Study 1: *N* = 46; Study 2: *N* = 55) were collected. However, due to partially missing data (*N* = 10) and other exclusion criteria (i.e., psychiatric disorders and left-handedness; *N* = 2) the final sample size comprises of 89 healthy young adults (Study 1: *N* = 36; Study 2: *N* = 53; age: *M* = 21.88; *SD* = 3.43; female: *N* = 57).

### Materials and Stimuli of the Behavioral Test Session

#### Berlin Intelligence Structure Test (BIS-T)

Participants’ numerical, verbal and figural intelligence was measured by the short version of the Berlin Intelligence Structure Test (Berliner Intelligenzstruktur-Test—BIS-T; Jäger et al., 1997). This test comprised 16 different tasks that either draw on the numerical (number of tests: 5), the verbal (number of tests: 6) or on the figural (number of tests: 5) component of intelligence. Additionally, each subtask can be assigned to four operational abilities, namely processing speed, memory, reasoning, and creativity. To assess numerical intelligence participants had to: (1) continue number series (reasoning); (2) cross out numbers in a matrix that were bigger by the factor of three in comparison to the preceding number (processing speed); (3) memorize pairs of digits (memory); (4) estimate the results of complex calculations (reasoning); and (5) to find different operands resulting in a given arithmetic solution (creativity). Tasks assessing verbal intelligence required participants to (6) fill in a missing letter resulting in a given word (processing speed); (7) list as many abilities a good salesman should not have (creativity); (8) complete analogies (reasoning); (9) decide whether a given statement represents a fact or an opinion (reasoning); (10) answer questions to a memorized text (memory); and (11) to cross out words that are meronyms of the preceding word (e.g., “year” preceded by the word “month”; processing speed). Finally, to assess figural intelligence participants had to (12) complete figural analogies (reasoning); (13) memorize marked buildings on a city map (memory); (14) design as many logos for a bike shop (creativity); (15) cross out “x” in an array of letters (processing speed); and (16) to complete figures (reasoning). For the subsequent analysis, we used sum scores for each intelligence component (potential maximum scores: numerical = 100; verbal = 128; figural = 181) that were based on raw scores (i.e., number of correct answers) of each subtask.

#### Arithmetic Fluency Task

Arithmetic competencies were assessed through a paper-pencil task developed by Vogel et al. (2017b) that is based on the French Kit Test (French et al., 1963). This test measures performance on simple and complex arithmetic problems. Participants were presented with 128 simple multiplications (i.e., consisting of two single-digit operands), followed by 64 simple additions (i.e., consisting of two single-digit operands), 128 simple subtractions (i.e., consisting of an operand <20 and a single-digit operand), 60 complex multiplications (i.e., consisting of a double-digit and a single-digit operand), 60 complex additions (i.e., consisting of three double-digit operands) and, finally, 60 complex subtractions (i.e., consisting of two double-digit operands). Participants were instructed to solve as many problems as possible within a given time. For solving simple arithmetic problems participants were given 90 s for each operation and 120 s for each operation type of the complex arithmetic problems. We then computed two scores indicating the numbers of correctly solved problems separately for each complexity level (i.e., simple vs. complex), where 320 was the potential maximum score for simple arithmetic problems and 180 for complex arithmetic problems.

#### Mathematics Test for Selection of Personnel (M-PA)

We assessed the subject’s performance in higher-order mathematics by using the short-version of the mathematics test for personnel selection (Mathematiktest für die Personalauswahl—M-PA; Jasper and Wagener, 2011). This test was developed to guarantee an optimal selection of suitable applicants based on their mathematical competencies. It is designed for teenagers and adults between 16 and 40 years who have at least a secondary school degree. The short version of the test comprises 31 complex mathematical problems presented as multiple-choice (MC) or open answer (OA) questions. Problems include fractions (3 OA), conversion of units (3 OA), exponentiation (7 OA), division with decimals (2 OA), algebra (1 MC), geometry (1 MC), roots (2 OA), and logarithm (7 OA). In total, participants had 15 min to solve the problems. For the subsequent analyses numbers of correctly solved problems were calculated (potential maximum score = 31).

#### Experimental Procedure

Behavioral data were collected in test sessions before the neuroimaging test sessions. Behavioral testing took place in laboratories of the Institute of Psychology at the University of Graz. One session took about 3 h, and participants were tested in small groups of a maximum of 12. After the greeting and general instruction participants were seated at a desk with a booklet, a computer screen and a keyboard on it. Desks were separated by partition walls, so that participants were able to work undisturbed. The booklet included a general instruction and all paper-pencil tests described in the “Materials and Methods” section. Additionally, the booklet included tests for measuring specific personality traits (i.e., the “NEO-Fünf-Faktoren Inventar—NEO-FFI” Borkenau and Ostendorf, 1993), the “German adaptation of the Abbreviated Math Anxiety Scale—AMAS-G” (Schillinger et al., 2018), the “Single-Item-Math Anxiety Scale—SIMA” (Núñez-Peña et al., 2014), a test measuring the attitude towards mathematics (Núñez-Peña et al., 2013, 2014), the “German Test Anxiety Inventory” (Prüfungsangstfragebogen, PAF; Hodapp et al., 2011), the “State-Trait Anxiety Inventory Trait scale—STAI-T” (Laux et al., 1981; Spielberger et al., 1983), and instructions for two computerized tasks (i.e., a single-digit multiplication task and an associative memory task), which participants also had to perform. The results of the latter tasks were not within the scope of the present study.

The order of tests was as following: BIS-T, M-PA, arithmetic fluency, computerized single-digit multiplication task, computerized associative memory task, NEO-FFI, PAF, attitudes towards mathematics, AMAS-G, SIMA, and STAI-T. Participants were instructed to work through the booklet page by page and to pause whenever they reached a page with a red stop sign. A trained experimenter took the time for all speeded tests (i.e., BIS-T: 45 min, M-PA: 15 min, arithmetic fluency: 10.5 min), and informed participants when they had to stop working on the respective test. At the end of the test booklet, participants were asked to fill out demographic questions according to their sex, age, field of study, and the mathematics grade in their final high school exam. In Austria, the grading system consists of a five-point scale, where 1 (“Sehr gut” – “very good”) is the best possible grade and 5 (“Nicht genügend” – “unsatisfactory”) is the lowest possible grade.

#### MRI Data Acquisition

Neuroimaging data were collected with a 3.0 T Siemens Skyra MRI scanner at the MRI Lab Graz. A 32-channel head coil and a Generalized Autocalibrating Partially Parallel Acquisitions (GRAPPA) sequence (TR = 1,950 ms, TE = 2.89 ms, 1 × 1 × 1 mm isotropic voxel resolution) was used to acquire the high-resolution T1-weighted anatomical images.

In addition to high-resolution T1-weighted anatomical images other neuroimaging data were collected in both studies: In study 1, functional and diffusion MRI (fMRI and dMRI) data were acquired. Participants had to perform three different tasks to measure the neural correlates of various cognitive processes. Results of the neurofunctional investigation are reported in Heidekum et al. (2019). In study 2 high-resolution T1-weighted anatomical images of participants were acquired within a training study that aimed to investigate the learning of arithmetic facts (i.e., multiplications). Participants underwent a 5-day multiplication fact training in which they intensively practiced a set of multiplications. Following the multiplication facts training, an MRI test session was conducted. In addition to the collection of anatomical data, functional imaging (i.e., task-based and resting-state activation data) as well as dMRI data were collected in this session. Since the behavioral and neurofunctional results of the training study are not within the scope of the present study they will be reported separately.

#### Analysis of Behavioral Data

Correlation coefficients were calculated to examine associations between all behavioral measurements. We calculated Spearman’s correlation coefficients for investigating associations with mathematics grade, whilst for the rest of the associations, Pearson’s correlation coefficients were calculated. *P*-values were Bonferroni corrected for 21 tested correlations (adjusted level of significance: *p*_Bonf_ < 0.00238). The normal distribution of each behavioral variable was verified by checking quantile-quantile plots and by calculating skewness and kurtosis. None of the variables significantly deviated from normality (i.e., *z*_Skewness_ > 1.96 or *z*_Kurtosis_ > 1.96).

#### Surface-Based Morphometry Analysis

SBM analyses were performed with the Computational Anatomy Toolbox (CAT12; Gaser and Dahnke, 2016), which is based on the Statistical Parametric Mapping (SPM, Welcome Department of Imaging Neuroscience, London, UK) software. In the first preprocessing step, T1-weighted anatomical images were normalized using “Diffeomorphic Anatomical Registration using Exponentiated Lie algebra” (DARTEL; Ashburner, 2007) and further segmented into GM, white matter (WM) and cerebrospinal fluid (CSF). In a next step, indices for cortical thickness (Dahnke et al., 2013), for cortical surface complexity (Yotter et al., 2011), for gyrification (Luders et al., 2006) and sulcal depth (Yotter et al., 2011) were calculated.

##### Cortical Thickness

Describes the distance between the inner surface (or boundary between GM and WM) and the outer surface (or boundary between GM and CSF; Dahnke et al., 2013). Cortical thickness, for instance, is an important biomarker for typical (O’Donnell et al., 2005) as well as atypical development (Thompson et al., 2004).

##### Fractal Dimensionality Index (Cortical Surface Complexity)

Characterizes the surface shape of the brain by quantifying the spatial frequency of gyrification and fissuration of the brain surface (Luders et al., 2004). More precisely, brain regions with a higher cortical surface complexity have a higher convolution level and therefore a larger surface area (Li et al., 2007; Yotter et al., 2011). Besides the investigation of age-related differences in brain structure (Madan and Kensinger, 2016), cortical complexity has also been successfully used to study differences in cognitive functions (King et al., 2010; Sandu et al., 2014).

##### Gyrification Index

Measures the regional curvature (i.e., convexity and concavity; Drury and Van Essen, 1997) of the brain and is defined as the ratio of the inner surface size to the outer surface size of an outer (usually convex) hull (Thompson et al., 1996; Luders et al., 2004, 2006). The gyrification index has typically been used to study group differences, such as between men and women (Luders et al., 2004) or between patients and controls (White et al., 2003; Shaw et al., 2012).

##### Sulcal Depth

Is based on the squared-transformed Euclidean distance between the central surface (average of the inner surface and the outer surface) and its convex hull (Lohmann, 1998; Yotter et al., 2011). Neuroanatomical studies using sulcal depth indices have shown, for instance, that it can account for differences in intelligence (Im et al., 2011; Yang et al., 2013).

Finally, full-width-at-half-maximum (FWHM) Gaussian kernels were used to smooth T1-weighted anatomical images. Following the matched-filter theorem, a 15.0 mm FWHM Gaussian kernel was applied for cortical thickness and a 20.0 mm one was used for folding measures (i.e., sulcal depth, gyrification, and cortical surface complexity).

In SPM we then performed second-level analyses to investigate whether structural variations in brain surface measures relate to individual differences in adults’ mathematical competencies. Multiple regression analyses for each mathematical competence measure (i.e., mathematics grade, simple arithmetic, complex arithmetic, higher-order mathematics, and numerical intelligence) were calculated separately. The multiple regression analyses were performed for each brain surface measurement (i.e., cortical thickness, cortical surface complexity, gyrification, and sulcal depth) and were controlled for the influence of verbal and figural intelligence as well as for age and sex (i.e., additional regressors of no interest). Statistical results of these whole-brain analyses are reported with family-wise error (FWE) corrected values at the cluster level (*p* < 0.05).

## Results

### Descriptive Analyses

In Table 1 means, standard deviations and ranges for all behavioral measurements are displayed.

**Table 1:** Descriptive statistics of all behavioral measurements (raw values).

	*N*	*M*	*SD*	Range
Mathematics grade^1^	89	2.47	0.96	(1–5)
Simple arithmetic	89	152.74	47.56	(52–246)
Complex arithmetic	89	47.63	19.28	(13–92)
Higher-order mathematics	89	21.20	5.49	(10–31)
Numerical intelligence	89	44.45	15.03	(12–87)
Verbal intelligence	89	92.11	16.14	(42–126)
Figural intelligence	89	84.12	11.97	(54–112)

*Note: ^1^Characteristics of the Austrian grading system: 1.00 = Very Good, 2.00 = Good, 3.00 = Satisfactory, 4 = Adequate, 5 = Unsatisfactory; Abbreviations: *M* = Mean; *SD* = Standard Deviation*.

### Correlation Analyses

Table 2 displays the correlations between all behavioral measurements. Almost all experimental measures that were thought to measure the same underlying component (i.e., mathematical cognition)—i.e., mathematics grade, simple arithmetic, complex arithmetic, higher-order mathematics and numerical intelligence—were significantly correlated with each other. The strongest association was found between performance on simple and complex arithmetic problems (*r* = 0.810, *p* < 0.001).

**Table 2:** Correlation coefficients between the behavioral measurements.

	(1)	(2)	(3)	(4)	(5)	(6)	(7)
(1) Mathematics grade	-						
(2) Simple arithmetic	−0.282	-				
(3) Complex arithmetic	−0.358*	0.810*	-				
(4) Higher-order mathematics	−0.356*	0.388*	0.471*	-		
(5) Numerical intelligence	−0.346*	0.681*	0.523*	0.379*	-	
(6) Verbal intelligence	−0.175	0.428*	0.454*	0.139	0.461*	-
(7) Figural intelligence	−0.183	0.343*	0.325*	0.165	0.420*	0.370*	-

*p < 0.00238 (Bonferroni corrected *p*-value).

For the mathematics grade, we found negative correlations with performance on complex arithmetic problems (*r* = −0.358, *p* < 0.001) as well as knowledge in higher-order mathematics (*r* = −0.356, *p* < 0.001) and numerical intelligence (*r* = −0.346, *p* < 0.001). This means that individuals with a better mathematics grade (associated with a smaller number) had better scores on experimental tests measuring mathematical abilities.

Additionally, both simple and complex arithmetic was positively correlated with verbal and figural intelligence. However, there was no significant correlation of mathematics grade with verbal and figural intelligence and no significant correlation of higher-order mathematics with verbal and figural intelligence. Finally, as can be expected, all three intelligence components (i.e., numerical, verbal and figural intelligence) were positively correlated with each other.

### Structural Correlates of Mathematical Competencies

Multiple linear regression analyses were performed to investigate whether structural variations in brain surface measures (i.e., cortical thickness, cortical surface complexity, gyrification, and sulcal depth) relate to individual differences in adults’ mathematical abilities (i.e., mathematics grade, simple arithmetic, complex arithmetic, higher-order mathematics and numerical intelligence). The results are as followed:

Cortical thickness: no significant negative or positive associations were found.

**Figure 1:**
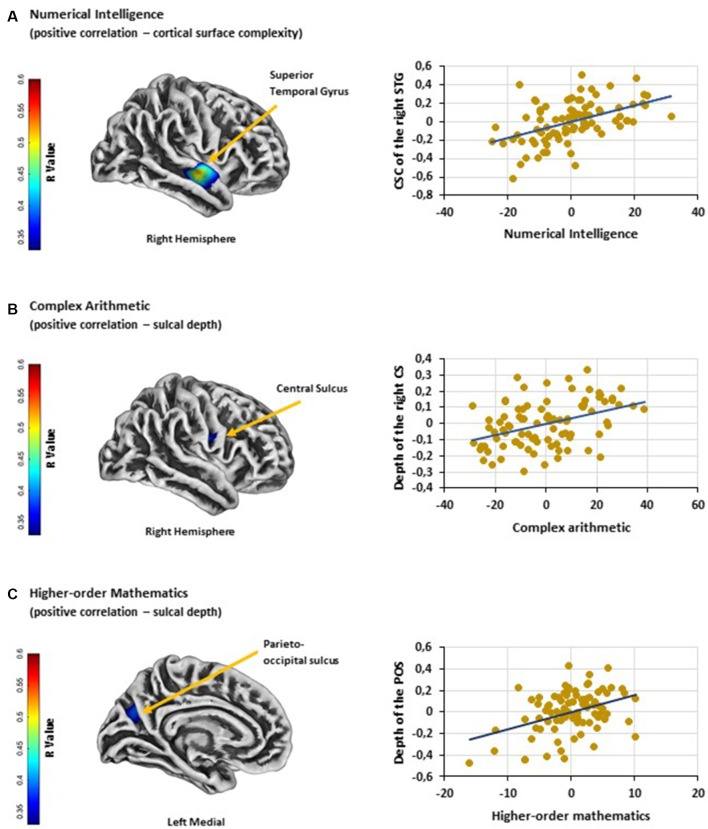
**(A)** Significant positive association between numerical intelligence and cortical surface complexity of the right STG. **(B)** Significant positive association between performance on complex arithmetic problems and the depth of the right CS. **(C)** Significant positive association between higher-order mathematics competence and the depth of the left POS. Scatter plots showing partial correlations between **(A)** numerical intelligence and cortical surface complexity of the right STG, **(B)** performance on complex arithmetic problems and the depth of the right CS and **(C)** higher-order mathematics competence and the depth of the left POS. Colorbar: correlation coefficients (R-value). Abbreviations: STG, superior temporal gyrus; CSC, cortical surface complexity; CS, central sulcus; POS, parieto-occipital sulcus.

Cortical surface complexity: analyses revealed that numerical intelligence was positively associated with the cortical surface complexity of the right superior temporal gyrus [MNI (x, y, z): 60, −9, −7; *p* < 0.05 FWE cluster corrected; *k* = 287; Figure 1A]. No significant negative associations were found.

Gyrification: no significant negative or positive associations were found.

Sulcal depth: results showed a positive association between performance on complex arithmetic problems and the depth of the right central sulcus [MNI (x, y, z): 54, −9, 23; *p* < 0.05 FWE cluster corrected; *k* = 141; Figure 1B]. Additionally, multiple linear regression analysis revealed a positive relationship between adults’ competence in higher-order mathematics and the depth of the left parieto-occipital sulcus [MNI (x, y, z): −18, −70, 29; *p* < 0.05 FWE cluster corrected; *k* = 161; Figure 1C]. No significant negative associations were found.

## Discussion

The current study is the first to investigate the associations between variations in brain surface structure (i.e., cortical thickness, cortical surface complexity, gyrification index, and sulcal depth) and individual differences in a broad range of mathematical abilities (i.e., mathematics grade, performance on simple and complex arithmetic problems, higher-order mathematics and numerical intelligence) within a large sample of typically developed adults. Analyses revealed three brain regions that were associated with individual differences in mathematical abilities: (1) the cortical surface complexity of the right superior temporal gyrus was positively related to numerical intelligence; (2) the depth of the right central sulcus was positively associated with individual’s ability to solve complex arithmetic problems; and (3) the depth of the left parieto-occipital sulcus showed a positive relationship with the individual difference in performance on higher-order mathematics. Although SBM, as a tool to assess local brain morphology, does not allow any direct assumption of local brain function, this study supports the idea that these brain regions play a role in mathematical thinking. Surprisingly, we did not find any associations between adults’ mathematical abilities and the neuroanatomical differences of previously reported brain regions, such as the IPS (Li et al., 2013; Lubin et al., 2013; Price et al., 2016). Conversely, all three brain regions that were observed in the present study are not typically associated with mathematics-related cognitive abilities. Despite these unexpected findings, the following sections will be an attempt to link functional properties to the observed cortical regions. Since the present study can only speculate on the functional role of these regions, further research (applying SBM for investigating brain-behavior relationships in the field of mathematical cognition) is needed.

First, our analyses revealed a positive relationship between the cortical surface complexity of the right superior temporal gyrus [MNI (x, y, z): 60, −9, −7] and individual differences in numerical intelligence. Surface complexity was defined by the fractal dimensionality index, which quantifies the spatial frequency of gyrification and fissuration of the brain surface (Luders et al., 2004). Thus, the current finding indicates that in individuals with higher numerical intelligence the right superior temporal gyrus shows a higher convolution level and therefore a larger surface area. Cortical complexity has been successfully used to investigate age-related differences in brain structure (Madan and Kensinger, 2016) and, more importantly, to study differences in cognitive functions (King et al., 2010; Sandu et al., 2014). Unfortunately, the scarce neuroanatomical studies on the correlates of the right superior temporal gyrus are typically based on comparisons between various groups of patients and controls (e.g., schizophrenia: Honea et al., 2008; Nenadic et al., 2010; depression: Mak et al., 2009; Peng et al., 2011). Due to the methodological differences of these studies, they do not allow an interpretation of the current findings. Similarly, the results of neurofunctional studies are very heterogeneous. The right superior temporal gyrus has been implicated in several cognitive processes, such as spatial awareness (Karnath, 2001), emotion processing (Narumoto et al., 2001) or the detection of biological motion (Akiyama et al., 2006). Interestingly, previous work (Jung-Beeman et al., 2004) has also linked activity in the right superior temporal gyrus to creative problem solving, as it might be responsible for binding and accessing various types of available conceptual representations (Shen et al., 2017); cognitive processes that might also be important for problems in a numerical intelligence test. Besides these findings on domain-general cognitive functions, some studies have found activity in this region to be related to mathematical cognition (Fehr et al., 2008; Simos et al., 2008; Andres et al., 2011, 2012; Kucian et al., 2011; Gullick et al., 2012). Since the numerical intelligence test used in the current study required the correct application of arithmetic operations (i.e., continuing number series; crossing out numbers in a matrix that were bigger by the factor of three in comparison to the preceding number; finding different operands resulting in a given arithmetic solution), it is interesting that the majority of these studies (Fehr et al., 2008; Simos et al., 2008; Andres et al., 2011, 2012) has linked activation within this region to arithmetic problem-solving. For instance, in an fMRI block design, Andres et al. (2011) instructed a group of young males to multiply single Arabic digits (between 3 and 9) with, or subtract them from, a predefined digit. By contrasting multiplication problems with subtractions, the authors observed increased activity in right superior temporal areas (among other brain regions), which they related to the storing of semantic knowledge of arithmetic problems. Additionally, Fehr et al. (2008) investigated fMRI activation patterns during mental addition, subtraction, multiplication, and division in young adults. In contrast to Andres et al. (2011), greater right-hemispheric superior temporal activation was found by contrasting complex with simple division, which might have reflected the usage of procedural strategies for problem-solving. Accordingly, even though these findings are based on neurofunctional studies and they relate activity within the right superior temporal gyrus to different arithmetic strategies, they are in line with the result of the current study. Nevertheless, additional research is needed to confirm and to further specify the observed brain-behavior relationship.

Second, our results also revealed a positive correlation between the depth of the central sulcus [MNI (x, y, z): 54, −9, 23] and adults’ ability to solve complex arithmetic problems. Previously, sulcal depth indices (e.g., sulcal fundi, lines or pits) have been successfully implemented to study the neuroanatomical correlates of various cognitive mechanisms. For instance, variations in the properties of sulci can account for individual differences in intelligence (Im et al., 2011; Yang et al., 2013), and abnormalities in specific sulci, such as the IPS or the superior temporal sulcus, have been observed in different groups of atypically developed individuals (e.g., dyslexia: Steinbrink et al., 2008; Richlan et al., 2013; dyscalculia: Rotzer et al., 2008; Price et al., 2016). Morphological changes of the central sulcus, on the other hand, have been associated with neurodegenerative diseases, such as multiple sclerosis (Pagani et al., 2005; Ceccarelli et al., 2008). However, the central sulcus also acts as an extension of its adjacent areas, namely the pre- and postcentral gyrus. Whereas the precentral gyrus is involved in eye movements (Corbetta et al., 1998; Anderson et al., 2007), postcentral activations have been linked to grasping (Simon et al., 2002)—both processes are commonly involved in cognitive tasks that include visually presented stimuli. For that reason, it is not surprising that these brain areas have also been found to play a role in numerical and calculation tasks (for a meta-analysis see Arsalidou and Taylor, 2011). For instance, Kaufmann et al. (2008) showed that activation patterns within these regions were related to non-symbolic numerical as well as spatial processing. And conversely, Kesler et al. (2006) have linked pre- and postcentral activations to the use of arithmetic strategies such as subvocalization and finger counting. In the present study, we observed a relationship between performance on complex arithmetic problems and the depth of the central sulcus. As the central sulcus acts as an extension of the pre- and postcentral gyrus, we, therefore, conclude that it might affect its adjacent mathematic-related regions.

Finally, the results of our SBM analyses revealed a positive association between the depth of the parieto-occipital sulcus [MNI (x, y, z): −18, −70, 29] and adult’s higher-order mathematical competence. The parieto-occipital sulcus lies between the posterior parietal and occipital cortices. This brain region is a crucial node of the dorsal visual stream and is, therefore, involved in visuospatial processing that support spatial navigation and goal-directed actions (Caminiti et al., 1999; Hutchison et al., 2015; Tosoni et al., 2015; Richter et al., 2019). However, the parieto-occipital sulcus also separates the cuneus from the precuneus and lies in the direct vicinity of the IPS. Whereas the cuneus is associated with visual information processing (Vanni et al., 2001), the latter brain regions (i.e., precuneus and IPS) were found to contribute to higher neurocognitive functions, such as attention (Corbetta and Shulman, 2002; Rosen et al., 2015), working memory (Barton and Brewer, 2013; Bray et al., 2015) or memory retrieval (Hebscher et al., 2019; Heidekum et al., 2019). Moreover, the precuneus and the IPS are often found to be involved in mathematical cognition (Dehaene et al., 2003; Arsalidou and Taylor, 2011). In particular, previous studies in the field of mathematical cognition have related brain activity in the precuneus to mental calculation (i.e., addition, multiplication, and subtraction; for a recent meta-analysis see Arsalidou and Taylor, 2011) and IPS activation to the representation and manipulation of numerical magnitude (Dehaene et al., 2003; or for a recent meta-analysis see Sokolowski et al., 2017). For instance, greater activation in the precuneus is often observed when arithmetic problems are solved by procedural strategies, rather than by memory retrieval (Delazer et al., 2005; Fehr et al., 2008). And, IPS activity is typically found whenever participants explicitly or implicitly compare the magnitude of numerals or dot-arrays (Pinel et al., 2001; Ansari et al., 2005; Vogel et al., 2017a; Wilkey et al., 2017). Interestingly, the functional role of the IPS within mathematics has also been confirmed by neurostructural studies (Isaacs et al., 2001; Rotzer et al., 2008; Rykhlevskaia et al., 2009; Ranpura et al., 2013) showing reduced GM in children with dyscalculia. In general, these studies highlight the importance of parietal brain regions in mathematical thinking, which is in line with the result of the current study showing an association between the parieto-occipital sulcus and higher-order mathematics.

Taken together, the current study highlights the potential of SBM as an alternative to classical VBM for investigating brain-behavior relationships in the domain of mathematical cognition. Associations were found between: (1) the cortical surface complexity of the right superior temporal gyrus and numerical intelligence; (2) the depth of the right central sulcus and adults’ ability to solve complex arithmetic problems; and (3) the depth of the left parieto-occipital sulcus and adults’ higher-order mathematics competence. Although two out of three brain regions (i.e., central sulcus and parieto-occipital sulcus) lie in intermediate vicinity of cortical structures typically associated with numerical and mathematical cognition, none of these brain regions were observed in previous studies. This discrepancy could be due to the methodological approach of the current study: In contrast to previous work, the present study studied linear brain-behavior relationships in a large sample of typically developed adults, whereas previous studies are mainly based on group comparisons between children with and without mathematical learning disabilities. For that reason, our results suggest that in typically developed individuals different neuroanatomical structures might become important when it comes to the processing of numerical and mathematical information. Finally, the current study shows that the depth of sulci could be a good index to investigate brain-behavior relationships. Nevertheless, future studies implementing SBM in different groups of individuals are needed.

## Data Availability Statement

The datasets generated for this study are available on request to the corresponding author.

## Ethics Statement

The studies involving human participants were reviewed and approved by the ethics commitee of the University of Graz. The patients/participants provided their written informed consent to participate in this study.

## Author Contributions

AH: conceptualization, data curation, formal analysis, writing—original draft. SV: conceptualization, formal analysis, funding acquisition, supervision, writing—original draft, writing—review and editing. RG: conceptualization, funding acquisition, project administration, supervision, writing—original draft, writing—review and editing.

## Conflict of Interest

The authors declare that the research was conducted in the absence of any commercial or financial relationships that could be construed as a potential conflict of interest.
